# Failing to Succeed: Advancing Mechanistic Understanding of Implementation Strategies Through Retrospective and Prospective Use of Causal Pathway Diagrams

**DOI:** 10.21203/rs.3.rs-7482638/v1

**Published:** 2025-09-23

**Authors:** Kirsten Austad, Kathryn L Fantasia, Arpan Mohanty, Kayla C. Jones, Nicholas A. Bosch, Mari-Lynn Drainoni

**Affiliations:** Boston University Chobanian & Avedisian School of Medicine / Boston Medical Center; Boston University Chobanian & Avedisian School of Medicine / Boston Medical Center; Boston University Chobanian & Avedisian School of Medicine / Boston Medical Center; Boston University; Boston University Chobanian & Avedisian School of Medicine / Boston Medical Center; Boston Medical Center/Boston University Chobanian & Avedisian School of Medicine

**Keywords:** Causal pathway diagrams, implementation strategy, realist evaluation, metabolic associated steatotic liver disease

## Abstract

**Background:**

Implementation strategies often fail to achieve their intended outcomes, yet limited methodological guidance exists for systematically understanding why strategies fail or how to adapt them for new contexts. Causal pathway diagrams (CPDs) are tools that map the mechanisms through which implementation strategies work. This study presents the first case study using CPDs both retrospectively to understand implementation failure in one context and prospectively to inform strategy adaptation for a new context.

**Methods:**

We conducted a case study of three failed implementation strategies—electronic best practice alert and clinical decision support, provider education, and clinical champion—designed to improve metabolic-associated steatotic liver disease (MASLD) screening in a weight management clinic. Using mixed methods data and realist evaluation principles, we constructed CPDs guided by three theoretical frameworks (i-PARIHS, Theory of Planned Behavior, and Technology Acceptance Model) and post-intervention qualitative interviews to understand failure mechanisms. We then applied insights from these CPDs alongside qualitative interviews with primary care providers to develop predictive CPDs for implementing adapted strategies in the primary care setting.

**Results:**

The retrospective CPDs revealed specific failure points not apparent during initial planning. The clinical decision support strategy failed because fundamental preconditions were unmet: only 54.2% of patients had recent laboratory results needed for automated calculations, and the alert placement misaligned with provider workflows. Provider education and clinical champion strategies were undermined by universal moderators including mistrust in the FIB-4 screening tool and competing clinical priorities. The prospective primary care CPDs identified necessary adaptations including replacing best practice alerts with integrated health maintenance reminders, adding content about MASLD complications and treatments to provider education, and selecting multiple champions to ensure adequate coverage.

**Conclusions:**

CPDs provide a systematic framework for transforming implementation failures into actionable insights for future intervention design. By distinguishing between preconditions necessary for mechanism activation and moderators that influence mechanism strength, CPDs enable precise identification of adaptation targets. The integration of realist evaluation principles with multi-theoretical CPD development offers a replicable methodology for mechanistic failure analysis and context-adapted implementation planning. This approach advances implementation science by moving beyond descriptive accounts toward rigorous understanding of how and why implementation strategies work across diverse settings.

## BACKGROUND

**Figure 1:**
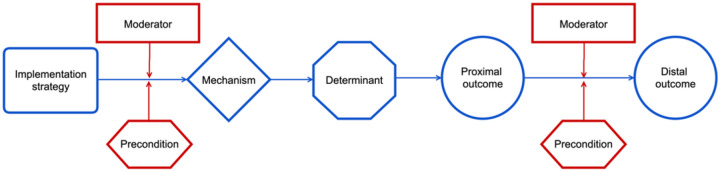
Anatomy of a causal pathway diagram (CPD). Blue represents the CPD stem and red the CPD leaves. Figure adapted from the ImpSciMethods.org Causal Pathway Diagram toolkit.(8)

Implementation strategies are the actions taken to promote the uptake of evidence-based interventions in real-world settings.(1) Recent scholarship in implementation science has increasingly shifted from cataloging and classifying implementation strategies toward a deeper focus on understanding the mechanisms through which these strategies produce their effects.(2–4) The causal pathway diagram (CPD) is a tool that can be used to understand how an implementation strategy achieves its intended impact.(5–8) CPDs link individual implementation strategies to measurable impacts (outcomes) by laying out the mechanism of action by which the CPD functions, the barriers and facilitators it targets (determinants), factors that influence its potency (moderators), and conditions that are necessary for it to function adequately (preconditions—Figure 1). To strengthen understanding of the mechanisms by which strategies function, existing theories and conceptual frameworks are drawn on when constructing CPDs.(7)

The logic of CPDs aligns closely with realist evaluation principles, which seek to understand not just whether an intervention works, but how, for whom, and under what circumstances that intervention achieves its intended outcomes.(9) Realist evaluation posits that implementation strategies work through underlying mechanisms that are triggered under specific contextual conditions to produce particular outcomes, articulated through Context-Mechanism-Outcome (CMO) configurations.(10, 11) This realist lens complements CPD methodology by providing a systematic approach to identifying the contextual factors that influence mechanism activation and outcome achievement.(12) The compatibility of these approaches is demonstrated in a recent publication that paired realist evaluation with mechanism mapping to retrospectively analyze why an intervention failed to improve treatment of gestational diabetes mellitus.(13) Although similar, CPDs differ from mechanism mapping by providing a more concrete, context-specific visualization that links implementation strategies to preconditions, mechanisms, moderators, and proximal outcomes, whereas mechanism mapping focuses more narrowly on hypothesized mechanisms of action at a higher level of abstraction.(14)

CPDs can be used prospectively to select, optimize, or evaluate implementation strategies as part of project planning. Recent work in implementation science has illustrated how CPDs can be developed to guide the selection and tailoring of strategies prior to implementation.(15, 16) While the developers of CPDs in implementation science have posited that they may also serve a retrospective diagnostic function, helping explain why an implementation strategy failed to produce desired outcomes, no published studies to date have illustrated this retrospective application in practice. This dual potential—to both diagnose implementation failures retrospectively and anticipate challenges prospectively—distinguishes CPDs from similar approaches like mechanism mapping and Theory of Change, which primarily focus on understanding how implementation strategies function but lack CPD’s precision regarding contextual influence.(14, 17–19)

In this case study, we illustrate both retrospective and prospective use of CPDs by developing two distinct sets of diagrams across two contexts. First, we used a realist evaluation-guided approach to construct CPDs retrospectively, using mixed methods data, to explain why a bundled implementation strategy failed to improve risk stratification for metabolic associated steatotic liver disease (MASLD) in a weight management clinic. The bundled strategy included (1) clinical decision support (CDS) and reminder in the form of a best practice alert (BPA), (2) a clinical champion, and (3) provider education—to improve rates of risk stratification for MASLD. Second, we used insights from these retrospective CPDs, combined with qualitative data on determinants of MASLD screening in primary care, to develop a second set of CPDs focused on anticipated future implementation in that new context to inform tailoring of the bundled strategy to the primary care context. Through this real-world, theory-driven example, we demonstrate how CPDs are valuable tools to (1) retrospectively diagnose reasons for implementation failure, (2) integrate mixed methods data to refine implementation strategies for a new setting, and (3) prospectively guide the development of a context-sensitive implementation plan.

### Case example: failed implementation strategy package for MASLD screening and detection

#### Quality Gap

MASLD affects 75% persons with obesity and is projected to rise in the coming decades. One in five persons with obesity have clinically significant fibrosis - a strong predictor of liver decompensation, hepatocellular carcinoma and adverse cardiovascular outcomes.

MASLD both lowers quality of life and poses the risk of life-threatening complications, such as cirrhosis and hepatocellular carcinoma.(20) It disproportionately impacts individuals from minoritized groups and those of low socioeconomic status, thereby contributing to inequities in downstream health outcomes. Effective therapies can prevent progression in those with clinically significant fibrosis, but because MASLD is asymptomatic until complications develop, early detection through screening is critical to improve outcomes. Unfortunately, MASLD is vastly underdiagnosed despite effective and non-invasive screening tools.(21, 22)

In 2021, the American Gastroenterological Association (AGA) released guidance that recommended a two-tier screening algorithm for individuals at risk for MASLD— i.e. those with type 2 diabetes, two or more cardiometabolic risk factors, or incidental finding of hepatic steatosis on imaging or elevated liver enzyme levels—to first undergo non-invasive serum testing, such as the Fibrosis-4 (FIB-4) score that can be calculated from age and three widely available laboratory tests (platelet count and liver enzymes AST and ALT).(23) Individuals with an FIB-4 score ≥ 1.3 should then undergo liver stiffness measurement with vibration-controlled transient elastography (VCTE) and those with FIB-4 score > 2.67 should also be referred to a hepatologist.

#### Project overview and context

The LINC (Leveraging Informatics for MASLD Care) intervention was designed to promote the uptake of MASLD risk stratification and screening into clinical practice in alignment with the AGA guidance. The project team included hepatologists and implementation scientists with both qualitative and quantitative expertise. The hospital system chosen for the study—an urban tertiary care academic medical center in New England that serves a diverse safety net population—provides care to a high proportion of low-income and minoritized patients with the majority covered by Medicaid and 50% patients identifying as Black and 25% of Hispanic descent.

Beginning in August 2021 we conducted a six-month pilot of the LINC intervention, which deployed three implementation strategies (described below) to improve rates of MASLD screening and evidence-based management in the hospital-based weight management clinic. The weight management clinic served a concentrated population of patients at high risk for MASLD and had a small, consistent group of providers regularly present in the clinic. The pilot concluded with a mixed methods evaluation that aimed to refine the implementation strategies before expansion to hospital-based primary care clinics and have been published elsewhere.(24) These projects were reviewed by the Boston Medical Center/Boston University Medical Campus Institutional Review Board and determined to be exempt.

#### Description of implementation strategies

To select implementation strategies, the planning team—comprised of clinicians within the health system—a priori generated a list of likely determinants of evidence based MASLD risk stratification based on their experience. The top drivers identified were low provider knowledge—of MASLD risk factors, options for non-invasive screening (e.g. FIB-4), interpretation of the FIB-4 score, and proper management of those at high risk for fibrosis (e.g. VCTE)—, competing clinical priorities, and provider inconvenience in calculating the FIB-4 score manually, which all led to low screening detection. The research team then selected and tailored three synergistic implementation strategies to improve uptake of MASLD risk stratification (full SPECIFY IT descriptions provided in Appendix 1):

Clinical decision support (CDS): The cloud-based technology Rimidi was selected to automatically calculate the FIB-4 score from laboratory results (platelets, AST, and ALT) as it could be integrated into the hospital system’s electronic medical record (EMR) Epic (Epic Systems, Madison Wisconsin). The FIB-4 score created a provider BPA displayed within the best practice setting of the EMR if the FIB-4 was elevated. If no lab results within the past six months were in the EMR at the time the visit was closed no FIB-4 score was calculated. If the FIB-4 was elevated (≥ 1.3), the CDS automatically suggested an order for VCTE and/or hepatology referral in line with AGA guidance, which providers could then select and accept (screen shot in Appendix 1). Data tracked for this implementation strategy included calculated FIB-4 score (continuous), if an alert was generated for elevated FIB-4 (binary), provider acknowledgement of BPA (binary), and if provider signed recommended order for VCTE or hepatology referral (binary).

Clinical champion: A provider from the weight management clinic was chosen by the research team based on their preexisting interest in MASLD and strong reputation amongst providers. The champion provided input on the content of the educational session, reinforced the educational messages regarding MASLD screening in the clinical space, and provided “at the elbow” support to clinicians (during their regular clinical time). They attended the research team monthly meetings to provide input on implementation. No specific metrics regarding the frequency of their interaction with frontline weight management staff were documented.

Provider education: An educational session targeting providers in the weight management clinic was designed by a hepatologist and included content on MASLD risk factors, non-invasive screening (FIB-4 and its interpretation), management of patients at high risk for liver fibrosis (VCTE, hepatology consult), and the LINC intervention including screen shots of the calculated FIB-4 score and populated orders recommended per 2021 AGA guidance. The presentation was delivered once via video conferencing software during a non-clinical time with opportunity for questions and answers and attendance was incentivized by providing continuing medical education credit for participation. We tracked the number of attendees at the session. The session was also recorded and distributed to providers for asynchronous review.

#### Post-pilot evaluation in weight management clinic

The post-pilot evaluation utilized a mixed methods approach, which is reported in full in a separate publication.(24) Quantitative data from the EMR during the six months after LINC implementation were compared to data from the six-month pre-intervention period. Briefly, we found that only 54.2% of patients (n = 2,513) had active lab results in the EMR to allow for calculation of the FIB-4 score prior to their visit. When available, the FIB-4 was calculated with 100% fidelity but most BPA firings recommending additional management were ignored by providers (n = 201, 85.9%). While there were more orders placed for VCTE (3 vs 0) and hepatology referral (7 vs 2) placed during the LINC implementation period compared to pre-implementation, the absolute number in both time periods was small.

We invited all providers in the weight management clinic to participate in semi-structured interviews guided by realist evaluation principles and two frameworks: i-PARIHS (integrated Promoting Action on Research Implementation in Health Services) and the Technology Acceptance Model (TAM).(25, 26) The interviews revealed that weight management providers felt it was within their scope of practice to screen patients for MASLD, however, their knowledge was low and skepticism about the reliability of FIB-4 was common. Regarding the implementation strategies, providers reported that while the educational session and clinical champion enhanced knowledge about MASLD risk stratification, they had persistent distrust in the FIB-4 score and competing clinical priorities that led to low adoption. Finally, while interviewees were positive about the value of an EMR-based clinical reminder, they identified several workflow misalignment issues that undermined its effectiveness. Many providers never accessed the BPA tab where the reminder was located, meaning they never encountered the alert. Among those who did use that BPA tab, they navigated there only at the end of the visit or after the patient had already left, making it impractical to discuss MASLD screening with the patient during the appointment.

#### Pre-implementation assessment in primary care

We planned to revise LINC based on lessons learned in the weight management context and expand it to the primary care setting in spring 2022. To this end, we conducted a sequential explanatory mixed methods study in the primary care clinics concurrent to the weight management pilot. Our findings from this assessment of the primary care context are published elsewhere.(27) Briefly, we first surveyed a diverse group of hospital-based primary care providers (PCPs) to assess their understanding of and perspective on MASLD. Surveys were used to recruit a convenience sample of these providers for semi-structured interviews which were guided by the theoretical domains framework (TDF) and TAM.

We found that PCPs felt that MASLD risk stratification was in their domain. However, we found a low level of knowledge about MASLD screening modalities (including FIB-4), low confidence in the ability of PCPs to manage it, and skepticism about ability of early detection of MASLD to change clinical outcomes given low knowledge of emerging therapies. Multiple barriers were felt to threaten the feasibility of MASLD screening in primary care, including the need to address other medical and social issues within the constraints of short appointments. Providers expressed positive attitudes toward EMR reminders as a strategy.

## METHODS

### Selection of guiding theories

We selected three complementary theoretical frameworks to guide the systematic construction of CMO and CPDs. To understand organization processes, we applied the integrated Promoting Action on Research Implementation in Health Services (i-PARIHS) framework, which conceptualizes successful implementation as a dynamic interaction between innovation characteristics, recipient factors, contextual influences, and facilitation processes.(28) We chose i-PARIHS because our clinical champion strategy leveraged facilitation, making it a good fit for understanding why these strategies underperformed.

However, i-PARIHS alone was insufficient to fully capture the nuances of individual-level behavioral change, which was critical given our ultimate outcome of altering individual provider screening behaviors. To address this, we incorporated the Theory of Planned Behavior (TPB), which specifically elucidates how attitudes, subjective norms, and perceived behavioral control shape behavioral intentions and subsequent actions.(29) TPB thereby provided a focused lens to identify and interpret individual provider-level barriers and motivators that i-PARIHS does not explicitly address. Recognizing that technology-mediated decision support was a key component of the intervention, we also included the TAM to capture challenges related to technology adoption and use.(25) TAM proposes that the perceived usefulness and ease of use affect users’ acceptance and integration of new technologies. Integrating TAM allowed us to dissect technological factors influencing implementation outcomes that were not explicitly encompassed by either i-PARIHS or TPB.

Together, these three theoretical frameworks provide a comprehensive multi-dimensional scaffold: i-PARIHS offers the organizational and contextual scaffolding; TPB illuminates provider-level behavioral mechanisms; and TAM addresses technological adoption factors. This integrative approach aligns with emerging consensus in implementation science emphasizing the value of multi-theoretical models.

### Construction of Context-Mechanism-Outcome configurations

We employed a sequential, mixed-methods approach to develop CMO configurations. First, the study team drafted preliminary CMOs based on the rationale underlying the selection of implementation strategies during the planning phase. Next, we analyzed quantitative data to pinpoint specific instances where the CDS strategy failed. The lead researcher then conducted an in-depth review of semi-structured interview transcripts, focusing on passages describing environmental, organizational, and individual contexts; psychological, social, or behavioral mechanisms activated by the implementation strategies; and observed or anticipated outcomes. A coding framework informed by three guiding theories was used to operationalize mechanisms and contextual moderators. Through constant comparison, the researcher identified patterns linking the activation or inhibition of mechanisms to proximal outcomes. These patterns were articulated as revised CMOs and refined through team discussions.

### Retrospective construction of CPDs

While other groups have used an intensive group “Deep Dive” approach to develop CPDs, characterized by intensive investigation and collective brainstorming, we adopted a streamlined process balancing individual and group input.(7) The lead author began by transforming the initial CMOs into the core causal pathway “stem” (illustrated in blue in Fig. 23). Next, moderators and preconditions—termed CPD “leaves”—along with proximal outcomes were added to construct comprehensive CPDs. Immersed in the guiding theories and models, the lead author made iterative revisions. A second author then reviewed the drafts, and both debriefed together, leading to further refinements. This cycle continued until no new insights emerged.

To validate the CPDs, we conducted member checking with a MASLD clinical expert and the project’s internal champion. An independent qualitative researcher, who was not involved in the original analysis, also reviewed the interview data to confirm the CPDs’ consistency with empirical findings. Any discrepancies arising during validation were resolved through team consensus.

Finally, using the finalized CPDs, the lead author performed a comparative analysis across the three implementation strategies, identifying CPD components common to multiple strategies versus those unique to individual interventions.

### Prospective construction of CPDs

To generate predictive CPDs for the primary care context using pre-implementation data, we applied a similar methodology. Starting with the CPDs developed retrospectively, the lead author immersed herself in primary care semi-structured interview transcripts to identify context-specific determinants influencing MASLD screening behavior.

To maintain theoretical consistency and rigor, we again utilized the three guiding theories—i-PARIHS, the Theory of Planned Behavior (TPB), and the TAM—to interpret qualitative findings and map new or modified determinants onto the CPD structure. The lead author reviewed each moderator and precondition from the weight management clinic CPDs, triangulating with primary care data to determine which factors were salient in the new setting and to identify new determinants unique to primary care.

Each CPD component—including preconditions, moderators, proximal outcomes, and mechanisms of action—was examined and adapted as needed to reflect contextual differences revealed by the qualitative data. Draft prospective CPDs were then circulated among MASLD content experts and three practicing primary care providers (PCPs) at the study site. Providers were asked to evaluate the plausibility of hypothesized pathways, identify missing contextual factors, and provide feedback on potential barriers or facilitators influencing the effectiveness of each strategy in primary care.

An independent qualitative researcher again reviewed the original qualitative data to verify alignment with the predictive CPDs. This feedback informed a final set of revisions, producing refined prospective CPDs that integrate empirical data and stakeholder input.

## RESULTS

### Constructing causal pathway diagrams: moderators and preconditions

**Figure 2:**
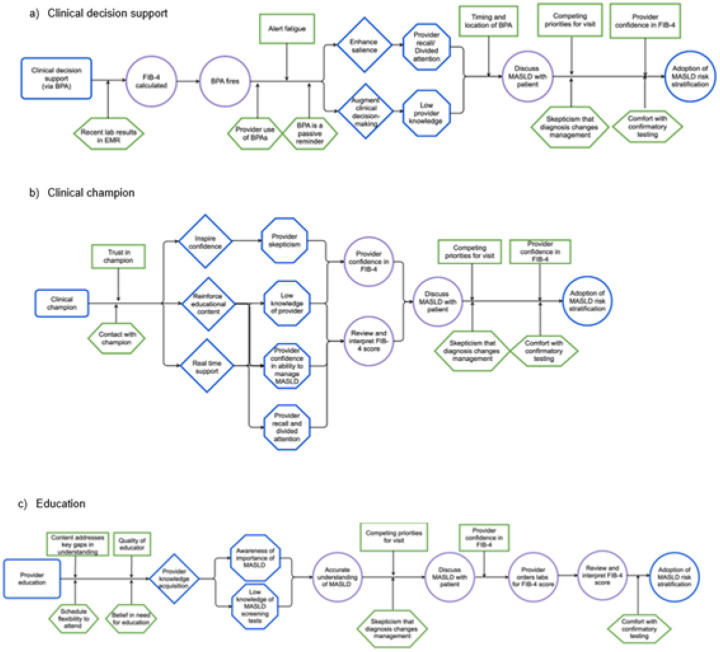
Causal pathway diagrams of LINC (Leveraging Informatics for MASLD Care) implementation strategies developed from lessons learned from failed implementation in weight management context. Figure A is for electronic medical record (EMR)-integrated clinical decision support and reminder, Figure B clinical champion, and Figure C provider education. The blue is the components of the stem based on causal logic in planning LINC. Purple are proximal outcomes identified during the pilot. Green are the leaves added based on post-implementation evaluation in the weight management clinic, including from qualitative interviews and additional insights from team debrief. The distal outcome is adoption of MASLD risk stratification, defined as ordering of vibration-controlled transient elastography (VCTE) or hepatology referral.

Figure 2 shows the CPDs generated after failed implementation in the weight management context. Table 2 summarizes the moderators and preconditions identified. Some of the moderators were likely to attenuate the impact of all three implementation strategies. First, deep mistrust in the FIB-4 score that some participants reported would make them less likely to respond to education, clinical champion, and the BPA. Similarly, competing patient and provider priorities during the visit could preclude uniform activation of the causal chain intended to improve MASLD screening. We identified two global preconditions that would preclude activation of the mechanism of action for all three implementation strategies. First, providers expressed discomfort with managing next steps following an elevated FIB-4 score (VCTE or referring to hepatology). Second, some providers questioned the value of diagnosing MASLD, expressing skepticism that it would alter clinical management.

We identified two key moderators that affected the effectiveness of the BPA strategy: alert fatigue and the timing and location of the BPA within the electronic health record. The BPA appeared in a section of the documentation that not all providers routinely accessed, and even among those who did, it was often viewed late in the workflow—typically after the patient encounter had ended. As a result, providers were unable to act on the MASLD screening recommendation during the visit without requiring follow-up contact or additional appointments.

Several preconditions emerged that may have contributed to the BPA’s failure to activate the intended mechanism of change. A critical one involved the FIB-4 calculation: the system could only generate a score if the patient had relevant laboratory results within the previous 12 months. This limitation meant that for approximately half of eligible patients, the BPA was unable to function as intended. Additionally, many weight management providers reported limited use with BPAs more broadly, reducing the likelihood that they would engage with it. Finally, the BPA was implemented as a passive reminder—meaning that providers were not required to respond to it—which further limited its impact on clinical behavior.

For the education implementation strategy, we uncovered two moderators. First, the effectiveness of the education depended on tailoring the content to address specific knowledge gaps among providers. Second, the quality of the educator will influence how well the implementation strategy’s mechanism is activated. We also identified two preconditions that could affect engagement with the implementation strategy and therefore its impact. Providers who believed they already had sufficient knowledge about MASLD were unlikely to participate in the educational session, reducing the potential for the strategy to influence practice. In addition, logistical barriers—such as scheduling conflicts or time constraints—could limit attendance and, in turn, its impact.

Qualitative interviews with providers highlighted trust in the clinical champion’s expertise as a key moderator influencing the effectiveness of the champion strategy. Providers were more likely to engage with and act on guidance when they viewed the champion as clinically knowledgeable. A critical precondition for this strategy was direct contact between providers and the champion. However, the champion reported being unable to offer real-time, “at the elbow” mentoring to all weight management clinic providers due to scheduling conflicts—specifically, limited overlap in clinical hours. This barrier disrupted the intended implementation pathway outlined in the CPD, in which mentoring from the champion was expected to enhance provider confidence and skills, ultimately leading to increased MASLD screening.

### Constructing causal pathway diagrams: proximal outcomes

To add proximal outcomes, we first integrated the quantitative process outcomes we collected at the end of the pilot—number of patients for whom a FIB-4 was calculated and the BPA fired—to the correct location along the causal pathway. Visually this reinforced that monitoring these outcomes during project rollout could have demonstrated that the cascade of downstream results intended would not be attained.

Several proximal outcomes were identified that were shared across more than one strategy (Table 3). Provider actions that could be easily measured to identify drop offs along the cascade include ordering of labs to calculate the FIB-4 and documenting interpretation and the result. While not so easily measured, knowing if the provider discussed the FIB-4 result with the patient would be valuable given its proximity in the causal chain to the desired behavior of the provider ordering VCTE or hepatology referral. Provider attitudes, specifically confidence in the FIB-4, could serve as a useful indicator of intention to use it. Lastly, for the implementation strategy of provider education, implementing some assessment of provider understanding of MASLD before and after could isolate the impact specific to that action.

### Prospective CPDs for primary care: moderators and preconditions

**Figure 3:**
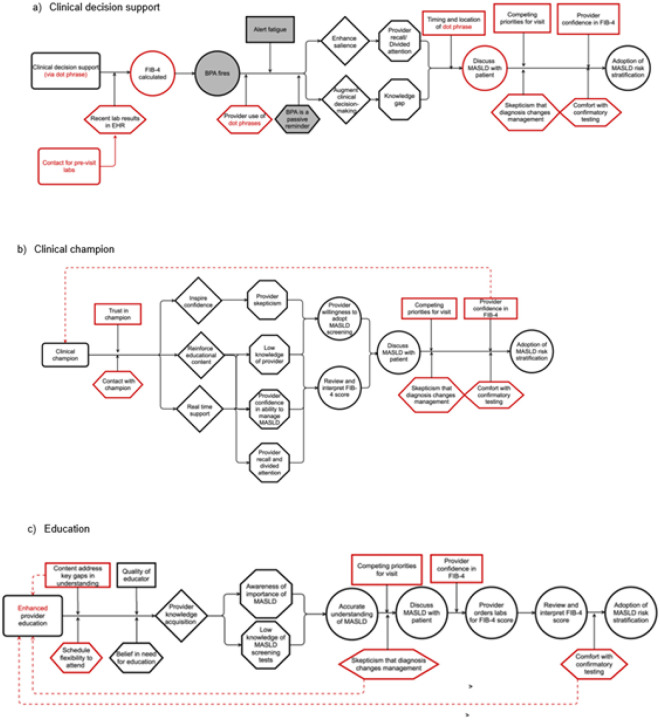
Revised causal pathway diagrams (CPDs) for LINC (Leveraging Informatics for MASLD Care) intervention adapted for future implementation in primary care setting. Figure A is for electronic medical record-integrated clinical decision support and reminder, Figure B clinical champion, and Figure C provider education. Black represents the components unchanged from the CPDs developed from implementation in the weight management context. Red represents changes made based on anticipated shifts in factors in the primary care context from qualitative interviews, construction of CPDs, and review by collaborators. Gray indicates components that are no longer relevant due need to significantly change the clinical decision support strategy. The distal outcome is adoption of MASLD risk stratification, defined as ordering of vibration-controlled transient elastography (VCTE) or hepatology referral. See Figure 1 for key regarding causal pathway diagrams depictions.

Figure 3 displays the CPDs for the primary care context. Participants in the primary care interviews were not supportive of MASLD risk stratification recommendations being deployed as a BPA. They acknowledged alert fatigue and felt that providers would ignore such recommendations in BPA form. Instead, they favored a text shortcut or template (known as a ‘dot phrase’) that could populate their note with the FIB-4 value and CDS, or integrating it into the Health Maintenance tab (a CDS module that tracks and manages adherence to preventive care services), which they often used. To address the lack of labs from which to calculate a FIB-4 the idea for a new implementation strategy of a pre-visit contact to patients with risk factors for MASLD to get labs drawn prior to the visit emerged from CPD construction. In the weight management context competing visit priorities was identified as a moderator. Similarly, primary care interview participants stressed that competing visit priorities would be a significant threat to success of the implementation strategy in primary care where appointments were shorter, and a broader range of topics need to be addressed. No specific ideas to address this moderator with a new implementation strategy emerged.

Using TPB to review findings from primary care interviews helped identify knowledge gaps and beliefs about capabilities to restructure the education implementation strategy for this context. Content domains that we identified that should be added to educational content include complications of MASLD (to address provider attitudes of low importance) and the existence of drug therapies for MASLD (to address provider attitude that diagnosis would not change management). To better promote progression from intention to screen to actual screening, participants identified specific skills including how to interpret a VCTE report that could aid providers.

Interviews also provided insights into how to strengthen the clinical champion implementation strategy. Lack of provider confidence in the FIB-4 was a major theme in the weight management context that was also echoed by participants in primary care. Better preparing the clinical champion to address distrust in FIB-4, such as by preparing rebuttals to common misconceptions, could enhance the implementation strategy. As primary care clinics are generally larger than the weight management context, selecting multiple clinical champions that are already identified as trusted peers and ensuring that time dedicated to providing “at the elbow” support to PCPs is another potential change.

### Predictive CPDs for primary care: outcomes

We reviewed all proximal outcomes mapped onto the CPDs. PCPs then provided feedback on the feasibility of measuring each outcome within their clinical context. To assess whether providers discussed MASLD with patients, PCPs suggested use of the dot phrase (to replace the BPA) as a proxy. A drop-down menu after the calculated FIB-4 could ask clinicians to document whether MASLD was discussed with patient, deferred to a future visit, or not addressed because the clinician disagreed with it signaling a high risk for MASLD.

To measure provider understanding, PCPs agreed that a brief survey on key points of understanding that can be administered at the beginning and end of the pilot. As provider trust in the FIB-4 was thought to be a critical moderator, we also explored how trust could be measured. Because no standardized scales exist, new tools are needed.

## DISCUSSION

There is a growing movement to understand the mechanisms behind implementation strategies to advance the field of implementation science.(2, 30) This study presents the first published case study demonstrating the simultaneous retrospective use of CPDs to understand implementation strategy failure and prospective application to inform tailoring them to a new context. We constructed CPDs using mixed methods data to examine why three implementation strategies failed in a weight management clinic, then leveraged these insights alongside qualitative data from PCPs to identify critical preconditions, moderators, and proximal outcomes for future implementation in this new context. Through this theory-driven approach, we demonstrate the value of CPDs for mechanistic failure analysis, mixed methods-informed strategy refinement across contexts, and precision implementation planning that systematically accounts for contextual factors.(25, 26, 29) Our work extends CPD methodology beyond its established prospective planning applications to provide a systematic framework for failure analysis and adaptive implementation.(15) Through the example of MASLD risk stratification, we demonstrate how CPDs can transform implementation failures into actionable insights that facilitate bringing implementation strategies to a different context.

The creators of CPDs are actively combining theory, empirical evidence, and expert opinion to develop CPDs for the most commonly used implementation strategies.(7) Our work builds on this foundation by demonstrating how CPDs can systematically dissect and learn from implementation failures to inform future efforts. Our analysis revealed critical failure points that were not anticipated during initial planning, particularly the cascade of preconditions that prevented mechanism activation. For example, the CDS strategy—delivered through a BPA—failed because fundamental preconditions operating at multiple levels were not met for a substantial proportion of cases. These included the availability of recent laboratory results for patients and proper alignment with provider workflow patterns.

By using CPDs to map these failure points, we can now anticipate which challenges may persist or emerge in a new context. This systematic understanding enables more informed discussions about whether implementation strategies can be tailored to address specific moderators and preconditions, ultimately facilitating more successful implementation in new settings.

Our analytical process began with realist evaluation’s CMO configurations, which provided a strong foundation for understanding how implementation strategies functioned in context.(9–11) However, our methodology demonstrates the distinct value that CPDs add beyond traditional realist approaches. While both frameworks examine mechanisms of action, CPDs excel at visualizing how multiple mechanisms can operate simultaneously within a single implementation strategy.(31) For instance, our provider education strategy operated through both knowledge acquisition mechanisms and confidence-building mechanisms, each with distinct pathways and potential failure points that would be difficult to capture in linear CMO configurations. Second, CPDs systematically distinguish between moderators that influence mechanism strength and preconditions that determine whether mechanisms can activate at all—a critical distinction for implementation planning that CMO configurations often conflate. Third, CPDs explicitly map proximal outcomes along the causal pathway, enabling identification of early indicators that can signal implementation success or failure before distal outcomes become apparent. The visual nature of CPDs synthesizes this complexity in ways that likely facilitate stakeholder understanding and collaborative refinement, though future research should empirically evaluate whether the visual representation enhances stakeholder engagement and decision-making compared to text-based frameworks.

**Figure 4:**
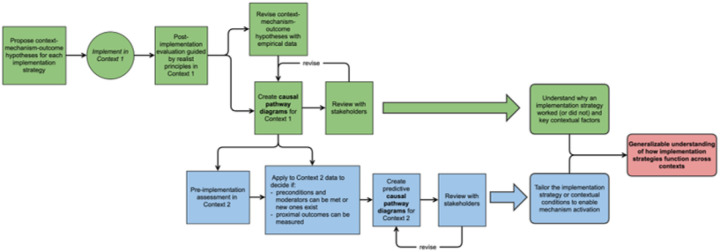
Methodological framework for leveraging CPDs and realist evaluation to conduct failure analysis and inform cross-context implementation strategy adaptation

Based on the experience described in this case, we propose a methodology for using CPDs within a realist framework for both retrospective and prospective applications (Fig. 4). Our approach with the MASLD example largely follows this methodology, with one important limitation: we conducted the pre-implementation assessment in primary care before creating the CPDs based on insights from the weight management implementation. Consequently, the primary care interviews were not designed to specifically explore the moderators, preconditions, and proximal outcomes that we later identified through the weight management experience.

Our CPDs revealed multiple proximal outcomes that could serve as early indicators of implementation success or failure. The ability to monitor FIB-4 calculation rates, BPA firing frequency, and provider acknowledgment patterns would have allowed for mid-course corrections during the initial implementation. This “fail fast” capability represents a significant advancement over traditional implementation approaches that rely primarily on distal outcomes.(6) The identification of measurable proximal outcomes also provides a roadmap for process evaluation in future implementations.

Our proposed methodology use of three complementary theoretical frameworks (i-PARIHS, TPB, and TAM) demonstrates how CPDs can serve as integrative tools that synthesize insights across different levels of analysis.(25, 26, 29) This multi-theoretical approach proved essential because implementation failures often result from misalignment across organizational, individual, and technological factors. i-PARIHS provided the organizational scaffolding, TPB illuminated individual provider decision-making processes, and TAM specifically addressed technology adoption challenges. This integration aligns with recent calls in implementation science to move beyond single-theory approaches toward more comprehensive explanatory models that better address the complexities of implementation contexts. (32)

The integration of realist evaluation principles significantly enhanced our CPD development process and vice versa. Realist evaluation’s emphasis on CMO configurations provided the analytical approach that facilitated CPD construction.(9–11) Its focus on mechanism heterogeneity—the recognition that identical implementation strategies can trigger different mechanisms across contexts—inspired the approach of anticipating challenges in the primary care context based on the evaluation from the weight management context. CPDs expanded upon what a realist evaluation alone could do primarily in depicting complexity, identifying moderators, preconditions, and proximal outcomes, and in depicting it in a more digestible way.

Our systematic comparison of CPD components across the weight management and primary care contexts provides a replicable framework for adapting implementation strategies across settings. Some components (such as provider education content) required substantial modification, while others (such as the basic mechanism of FIB-4 calculation) remained consistent. This analysis revealed that successful cross-context adaptation requires attention to both universal human factors (such as cognitive load and competing priorities) and setting-specific structural factors (such as workflow patterns and patient populations).

Several limitations should be acknowledged. First, the data used to construct both sets of CPDs originated from a single academic medical center, which may have limited the variability captured. Second, our primary care interviews were conducted concurrently with the weight management pilot, rather than being specifically designed to explore CPD components identified from the initial failure analysis. A more systematic approach would have explicitly tested the transferability of identified moderators and preconditions across settings. Third, although we involved clinical experts and an independent qualitative researcher in validating the CPDs, employing a more formal consensus process with a diverse group of key stakeholders could have strengthened the reliability of our findings. Likewise, engaging experts with prior experience using these implementation strategies to review the CPDs would have further enhanced methodological rigor.

## CONCLUSIONS

This study advances mechanistic understanding in implementation science by demonstrating how CPDs can systematically unpack the causal mechanisms through which implementation strategies fail and attempt to use that information moving forward in a new context. This moves beyond descriptive accounts to provide rigorous methodology for identifying specific mechanisms that link strategies to their intended effects, the contextual conditions that activate or inhibit these mechanisms, and the proximal indicators that signal mechanism functioning. Our integration of realist evaluation principles with theory-driven CPD development addresses a fundamental challenge in implementation science: understanding why strategies that work in one setting often fail when transferred to another. This mechanistic precision enables researchers to develop testable hypotheses about mechanism activation and identify necessary preconditions and moderators that must be considered in a new implementation setting to inform tailoring to strategies to context. This is essential for building cumulative knowledge and evolving toward more personalized and context-adapted implementation approaches.

Future research should explore the generalizability of our approach across different clinical conditions and implementation contexts. Particularly valuable would be studies that prospectively design CPDs using our integrated theoretical approach and then test their predictive validity. Additionally, research is needed to develop standardized methods for measuring some of the psychological constructs identified as key moderators, such as provider trust in clinical algorithms.

## Supplementary Files

This is a list of supplementary files associated with this preprint. Click to download.

- Appendix.docx

## Figures and Tables

**Table 1:** Context-Mechanism-Outcome theories

	Pre-Implementation	Post-Implementation
Clinical decision support (CDS) via Best Practice Alert (BPA)	In settings where providers have many competing demands and would need to calculate the FIB-4 by hand, point of care reminders with automated calculation of FIB-4 will result in an increase in MASLD risk stratification	When the configuration of the BPA CDS does not align with provider workflows, the BPA does not actually remind clinicians during patient encounter and thus does not increase application of FIB-4 for MASLD risk stratification
Clinical champion	In settings where providers have low knowledge of risk stratification tools for MASLD, a clinical champion can improve their use by providers	When providers have distrust in a screening tool, a clinical champion unprepared to address distrust is insufficient to change provider behavior and increase its use
Education	In settings where providers have low knowledge of risk stratification tools for MASLD, education can improve their use by providers	When providers have distrust in a screening tool, education is insufficient to change provider behavior and increase its use

**Table 2:** Moderators and preconditions identified through construction of causal pathway diagrams from post-pilot qualitative and quantitative data

**Moderators**
Shared: Competing priorities during visit
Shared: Provider confidence in FIB-4
Clinical decision support: Pop up fatigue
Clinical decision support: Timing and location of BPA
Education: Content address key gaps in understanding
Education: Quality of educator
Champion: Trust in champion
**Preconditions**
Shared: Skepticism that diagnosis changes management
Shared: Provider comfort with confirmatory testing
Clinical decision support: Recent lab results in electronic medical record
Clinical decision support: BPA is a passive reminder
Clinical decision support: Provider use of BPA tab
Education: Schedule flexibility to attend
Education: Belief in need for education
Champion: Contact with champion

**Table 3:** Outcomes identified through construction of causal pathway diagrams

**Proximal Outcomes**
Shared: Discuss MASLD with patient
Shared: Provider orders labs for FIB-4 score
Shared: Review and interpret FIB-4 score
Shared: Provider confidence in FIB-4
CDS: FIB-4 calculated
CDS: BPA fires
Education: Accurate understanding of MASLD
**Distal Outcomes**
Adoption of MASL risk stratification

## Abbreviations

BPA: best practice alert
CDS: clinical decision support
CMO: Context-Mechanism-Outcome
CPD: causal pathway diagram
EMR: electronic medical record
FIB-4: fibrosis-4
i-PARIHS: integrated Promoting Action on Research Implementation in Health Services
LINC: Leveraging Informatics for MASLD Care MASLD = metabolic-associated steatotic liver disease
PCP: primary care provider
TAM: Technology Acceptance Model
TPB: Theory of Planned Behavior
VCTE: vibration-controlled transient elastography
